# Translation and Validation of Bristol Female Lower Urinary Tract Symptoms Questionnaire for Urinary Incontinence in Urdu

**DOI:** 10.7759/cureus.23564

**Published:** 2022-03-28

**Authors:** Ahmad Bashir, Sahar K Zuberi, Rimsha Khan, M Hammad Ather

**Affiliations:** 1 Urology, Aga Khan University Hospital, Karachi, PAK; 2 Basic Sciences, Aga Khan University Hospital, Karachi, PAK; 3 Urology, Department of Surgery, Aga Khan University Hospital, Karachi, PAK

**Keywords:** women’s health, urdu, validation, questionnaire, urinary incontinence

## Abstract

Background

In this study, we aimed to translate and validate the 19-item version of the Bristol Female Lower Urinary Tract Symptoms (Bristol FLUTS) Questionnaire in Urdu among women experiencing urinary incontinence at a tertiary care hospital in Karachi, Pakistan.

Methodology

A cross-sectional validation study was conducted in the urology clinic at Aga Khan University Hospital, Karachi, Pakistan, between April and September 2021. After forward and backward translation of the Bristol FLUTS questionnaire, content validation was done by six experts, followed by the administration of the questionnaire to 10 respondents in the pilot phase of the study. In total, 207 participants were approached to fill the final version of the translated questionnaire. Overall, 188 respondents filled out the questionnaire, including 94 women with urinary incontinence and 94 women from the community to test construct validity. Finally, 30 women with urinary incontinence were asked to fill the form again two weeks later to determine test-retest reliability. Cronbach’s alpha was employed to assess the internal consistency of the questionnaire.

Results

The questionnaire displayed good content validity for reliability (content validity index: 0.84) and clarity (0.89). The scores reported by cases were significantly higher than the controls in all but the sexual function domain, suggesting good construct validity. Cronbach’s alpha of 0.81 signified good internal consistency, and a Pearson’s coefficient of 0.993 (p < 0.001) comparing responses at baseline and after two weeks indicated good test-retest reliability.

Conclusions

The Urdu translation of the Bristol FLUTS is a valid and reliable questionnaire that can be used in the clinical setting.

## Introduction

Urinary incontinence is defined by the International Continence Society as “the complaint of any involuntary leakage of urine.” This condition is found in 20% of women, with an age-related increase in prevalence from 17% in women over the age of 20 years to 37.5% in women aged between 30 and 50 years and 38% in women over 60 years of age [1]. Despite such a high prevalence, only 25% of affected women seek medical help, and even fewer receive treatment [2]. In Pakistan, the prevalence of urinary incontinence is 11.5% in women which is independently related to early marriages, increasing age, and greater parity [3]. Furthermore, 44.4% of obese women between 15 and 44 years of age have been reported to suffer from urinary incontinence [4].

The cases of urinary incontinence are grossly underreported due to various factors, including a sense of shame, reduced health expectations, denial, gender of the doctor (women are often uncomfortable disclosing it to male doctors), elderly age, low severity of symptoms, and uncertainty about confidentiality by the primary healthcare provider [5-7]. This underreporting adversely affects the quality of life of affected individuals leading to physical as well as psychological harm. These detrimental effects include social isolation, loss of self-confidence, depression and anxiety, problems in sexual life, and limitations in physical activity [8]. Moreover, for Muslim women, ablution performed for prayers becomes void with urinary leakage which can be troublesome in daily life [4,7].

Incontinence is commonly assessed by physicians based on their experience and urodynamic studies with little emphasis on how bothersome it is for the patient. The Bristol Female Lower Urinary Tract Symptoms (FLUTS) questionnaire was developed to assess the impact of the symptoms of incontinence on the quality of life of individuals. The questionnaire has 19 questions graded over a five-point Likert scale. They are divided into three domains rating the extent of urinary incontinence, quality of life, and sexual function. These include four questions regarding the filling phase, three regarding voiding, five regarding urgency, two regarding sexual function, and five regarding the effect of these symptoms on the quality of life [9]. The questionnaire serves as a non-interventional, economical, and rapid assessment tool that can easily be self-administered in clinics [9]. It can help identify urinary incontinence and provide therapeutic options to those who feel uncomfortable disclosing this problem otherwise.

Urdu is the official national language of Pakistan spoken by almost 85.5 million people. This study aimed to translate and validate the Bristol FLUTS questionnaire for its use in evaluating the psychological impact of incontinence on affected women. The current practice involves standard history taking by an individual in the urology clinic, ranging from a qualified urologist to trainees including residents, interns, and medical students. We aim to incorporate this questionnaire into the urological history note to standardize information acquired from the patient irrespective of the retriever.

The objective of this study was to determine the validity and reliability of the Bristol FLUTS questionnaire translated in Urdu among women experiencing urinary incontinence at a tertiary care hospital in Karachi, Pakistan.

## Materials and methods

A prospective cross-sectional study was conducted over six months in the urology outpatient clinic at Aga Khan University Hospital (AKUH), Karachi, Pakistan. The validity and reliability of the Urdu translation of FLUTS were evaluated by the consecutive sampling of 207 participants. Out of the 207 participants who were given the questionnaire, 188 chose to participate in the study, making a total of 94 cases and 94 controls. The sample size was calculated on Pass 2011 software. Based on the construct validity of the article validating the first Bristol FLUTS, the minimum sample size needed was 207 with 10% inflation, with an anticipated difference of proportion of symptoms of 20% in women with symptoms of urinary incontinence and those with no history of urinary incontinence [9]. Before administrating the questionnaire, it was forward and backward translated.

Inclusion criteria included women aged 18 to 75 years who gave consent to participate in the study. In total, 94 women had a history of urinary incontinence for at least three months and 94 women had no history of urinary incontinence over the last three months. The diagnosis of urinary incontinence was confirmed by an expert urologist. Exclusion criteria included women with previously diagnosed psychiatric or neurological diseases, those who had already undergone treatment for incontinence, had education status below primary level, or did not understand Urdu.

Translation

Two native Urdu translators with English as their first foreign language were asked to individually translate the questionnaire into Urdu. The two translations were incorporated into one and labeled FLUTS-1. Two additional native English translators with Urdu as their first foreign language were asked to retranslate FLUTS-1 back into English. Any variations in retranslation were incorporated in FLUTS-1, labeling this version FLUTS-2. FLUTS-2 was sent to six urology faculty members with clinical experience of more than five years to comment on any revisions they felt needed to be made to the questionnaire to ensure better clarity and appropriateness of questions. This final document was labeled FLUTS-3. A pilot study of FLUTS-3 was conducted among 10 women.

The final questionnaire was administered to 94 women with urinary incontinence and 94 healthy women. Finally, 30 women with urinary incontinence were asked to fill the questionnaire again at their follow-up visit two weeks later.

Content validity

Six urology faculty at AKUH were interviewed to ensure that the questions were relevant and clear in assessing the disease using a four-point Likert scale for relevance and clarity. The sum of responses of each item in a domain was divided by the raters to obtain a domain-specific average content validity index (CVI) score. For cumulative CVI score, an average of domain-specific scores was obtained and scaled down to 0 (Likert score 1-2) and 1 (Likert score 3-4), where 0 denoted no agreement and 1 represented perfect agreement.

Construct validity

To assess construct validity, the responses of women with symptoms of urinary incontinence were compared to those with no symptoms of the disease. In total, 94 consecutive women with complaints of urinary incontinence from all urology clinics (cases) and 94 women from the community with no history of urinary incontinence in the last three months were recruited (controls). Females presenting to urology clinics are expected to be more symptomatic than those in the community, and thus, women outside of urology clinics were also recruited to compare the community and patient population. The community group comprised women attendants in the AKUH hospital waiting area. The means of scores of individual items were compared between these two groups.

Reliability

Test-Retest

Because patients with urinary incontinence are routinely seen on follow-up, they were the ones asked to again fill out the questionnaire two weeks later. Considering 10-20% of the sample size is considered adequate for assessment of reliability, 30 participants were approached [10,11]. The responses at baseline were correlated with those three weeks later to determine test-retest reliability.

Internal Consistency

To assess the internal consistency of the tool, Cronbach’s alpha was calculated for all the 19 items in the questionnaire.

Statistical analysis

Data was analyzed on SPSS version 23 (IBM Corp., Armonk, NY) and Microsoft Excel. CVI was calculated for relevance and clarity for all items in the questionnaire, and a CVI of >0.7 was considered acceptable. To test construct validity, multiple independent t-tests were used to assess if the tool could correctly differentiate between women with urinary incontinence and those without. Cronbach’s alpha was calculated for assessing the internal consistency of the tool, and a value >0.70 was considered to be a measure of good internal consistency. Test-retest was measured using the cumulative scores of the five domains, namely, filling, voiding, incontinence, sexual function, and quality of life, using Pearson’s correlation coefficient. P-values of <0.05 were considered statistically significant.

Ethical considerations

Institutional ethical review approval was sought (ERC: 2021-6045-17273). Written informed consent was obtained from participants, and they were given full autonomy to opt out of the study without any impact on their treatment.

## Results

After forward and backward translation, the questionnaire was assessed for content and construct validity. Thereafter, 207 participants were approached over six months (April to September 2021). However, only 94 cases and 94 controls (n = 188) fulfilled the inclusion criteria and filled out the questionnaire.

Content validity

Based on the feedback from six experts, the calculated CVI for 19 items was 0.84 for relevance and 0.89 for clarity (Table 1). When asked about the relevance of questions, the experts were in universal agreement (UA) with eight items while the individual score was ≤0.5 (less than 50% agreement) for the following two items: “Do you stop and start more than once while you urinate?” and “Do you leak urine when you are asleep?.” For clarity, experts were in UA with 12 items and only one question had an individual score of ≤0.5: “Do you have pain in your bladder?” There was minimal missing data (four unfilled questions in total, 0.1%), suggesting clarity in how the questions were phrased.

**Table 1:** Scores of individual items rated by experts on the basis of their relevance and clarity. CVI = content validity index

CVI score (0-1)	Relevance (n = 19); n (%)	Clarity (n = 19); n (%)
1	8 (42.1)	12 (63.1)
0.83	6 (31.6)	2 (10.5)
0.67	3 (15.8)	4 (21.1)
0.5	2 (10.5)	1 (5.3)

Construct validity

Every item was rated on a Likert scale of 1-4 by the cases and controls. Cases displayed statistically significant higher scores in all but the sexual function domain compared to the controls, confirming that the questions successfully differentiated between women with the disease and controls (Table 2).

**Table 2:** Comparison of scores of individual items of the questionnaire in women with urinary incontinence and those without any symptoms. SE = standard error; CI = confidence interval, * = significant (p < 0.05)

	Case (n = 94); mean ± SE	Control (n = 94); mean ± SE	P-value (CI)
Filling			
F1	3.20 ± 0.14	1.67 ± 0.09	<0.001 (1.20,1.86)*
F2	3.12 ± 0.16	1.11 ± 0.03	<0.001 (1.67,2.33)*
F3	2.63 ± 0.10	2.30 ± 0.07	0.007 (0.09,0.57)*
F4	2.16 ± 0.14	1.10 ± 0.03	<0.001 (0.78,1.35)*
Voiding			
V1	1.77 ± 0.11	1.00 ± 0	<0.001 (0.64,1.19)*
V2	1.91 ± 0.14	1.00 ± 0	<0.001 (0.54,0.99)*
V3	2.41 ± 0.16	1.01 ± 0.01	<0.001 (1.08,1.73)*
Incontinence			
I1	2.32 ± 0.14	1.05 ± 0.02	<0.001 (0.99,1.54)*
I2	2.41 ± 0.13	1.11 ± 0.03	<0.001 (1.04,1.58)*
I3	1.21 ± 0.07	1.00 ± 0	0.005 (0.07,0.36)*
I4	2.53 ± 0.17	1.47 ± 0.08	<0.001 (0.70,1.43)*
I5	1.29 ± 0.09	1.00 ± 0	0.001 (0.11,0.46)*
Sexual function			
S1	1.03 ± 0.02	1.05 ± 0.23	0.473 (-0.08,0.04)
S2	1.19 ± 0.07	1.05 ± 0.23	0.067 (-0.01,0.29)
Quality of life			
Qol 1	2.94 ± 0.13	1.33 ± 0.07	<0.001 (1.32,1.89)*
Qol 2	2.37 ± 0.14	1.05 ± 0.02	<0.001 (1.04,1.60)*
Qol 3	3.60 ± 0.12	1.27 ± 0.05	<0.001 (2.07,2.58)*
Qol 4	2.99 ± 0.11	1.19 ± 0.04	<0.001 (1.56,2.03)*
Qol 5	3.45 ± 0.08	1.33 ± 0.07	<0.001 (1.92,2.32)*

Reliability

Internal Consistency Reliability

The total measured Cronbach’s alpha for the 19 items of the questionnaire was 0.81, suggesting good internal consistency. Cronbach’s alpha for the sum of each individual domain was also calculated and displayed good internal consistency in all domains except sexual function (CVI = 0.37). CVI scores for the rest of the domains were 0.55 for filling, 0.82 for voiding, 0.63 for incontinence, and 0.84 for quality of life.

Test-Retest Reliability

This measures reproducibility and constancy. The same questionnaire was administered to 30 women with urinary incontinence twice at an interval of two weeks when they visited the clinic for follow-up. When the total scores of responses two weeks apart were compared, Pearson’s r was 0.993 (p < 0.001), showing a strong correlation. Every domain was also independently analyzed (Table 3). The correlation between the responses at the two-week interval was statistically significant, suggesting that the tool is reliable.

**Table 3:** Correlation between the severity of symptoms at baseline and two weeks later.

	Pearson’s r	P-value
Filling	0.987	<0.001
Voiding	0.995	<0.001
Incontinence	0.986	<0.001
Sexual function	1.000	<0.001
Quality of life	0.978	<0.001

## Discussion

Bristol FLUTS is a comprehensive questionnaire that aims to gauge the severity of symptoms of urinary incontinence in women and to what extent they affect the quality of life of women experiencing them. It is also used to assess treatment outcomes. It comprises 19 questions divided into five domains [9]. It has previously been translated and validated in the Persian language [11].

Another questionnaire, International Consultation on Incontinence Questionnaire Female Lower Urinary Tract Symptoms (ICIQ-FLUTS), has been derived from Bristol FLUTS and has been widely translated and validated in different languages [12-16]. The only questionnaire addressing urinary incontinence that has been validated in Urdu is the Urogenital Distress Inventory (UDI-6) [17]. UDI-6 is a six-item questionnaire that assesses the impact of urinary incontinence on the daily life of women. However, due to its brevity, it does not address symptom severity and response to treatment, making Bristol FLUTS a more comprehensive tool for evaluating urinary incontinence.

Considering a CVI of 0.84 for relevance and 0.89 for clarity and only four missing values (0.1%) in our study, the content validity of Bristol FLUTS was very good and the questions were relevant and easily comprehensible. This is comparable to previously reported missing values ranging between 0.5% and 2% [9,11].

The construct validity was determined by grouping the responses of the symptomatic and general populations. The questionnaire successfully differentiated between the two as the general population reported significantly lower symptoms compared to those experiencing urinary incontinence, except for the questions regarding experiencing pain in the bladder, leaking urine during sleep, and urinary incontinence impairing sex life. This is similar to the results of the parent study that reported a higher percentage of most symptoms in the clinical population [9].

Internal consistency calculated using Cronbach’s alpha for all the 19 items was 0.81 which suggests good internal consistency. This lies between previously reported Cronbach’s alpha values ranging between 0.78 and 0.83 [9,11]. The test-retest reliability was measured using Pearson’s r. Total Pearson’s r of 0.99 is much higher than that reported previously, with one study reporting 0.77 and another reporting values ranging from 0.86 to 0.90.

One of the limitations of this study is that it was a single-center study.

## Conclusions

The Urdu translation of the Bristol FLUTS displayed very good validity and reliability and can be incorporated in the history taking of women presenting with urinary incontinence.

## Figures and Tables

**Figure 1:**
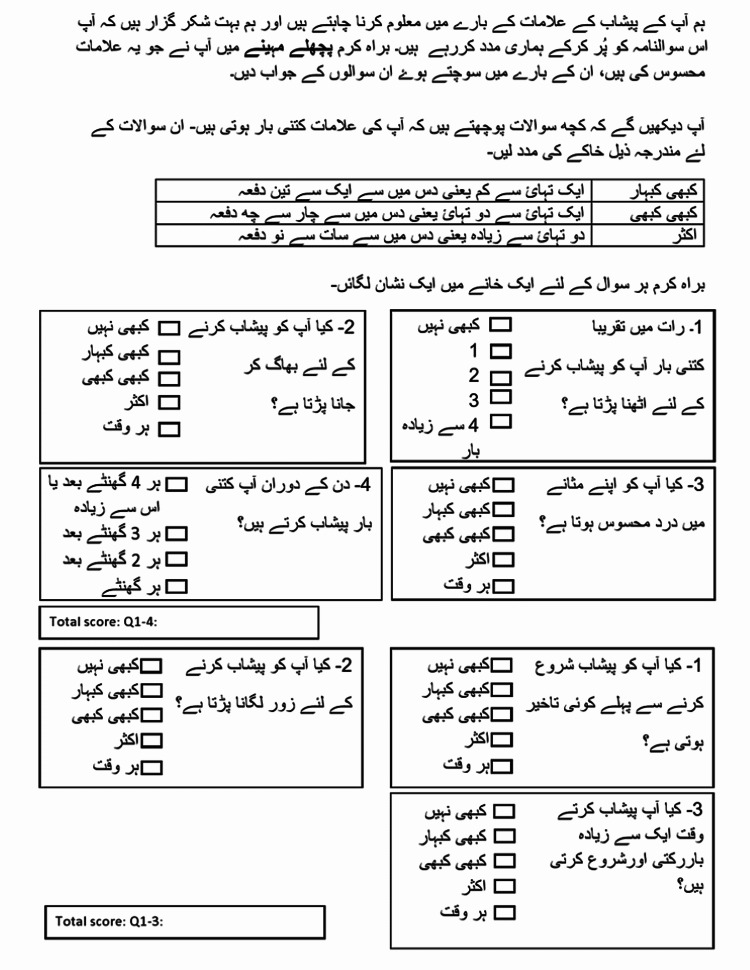
Urdu questionnaire: page 1.

**Figure 2:**
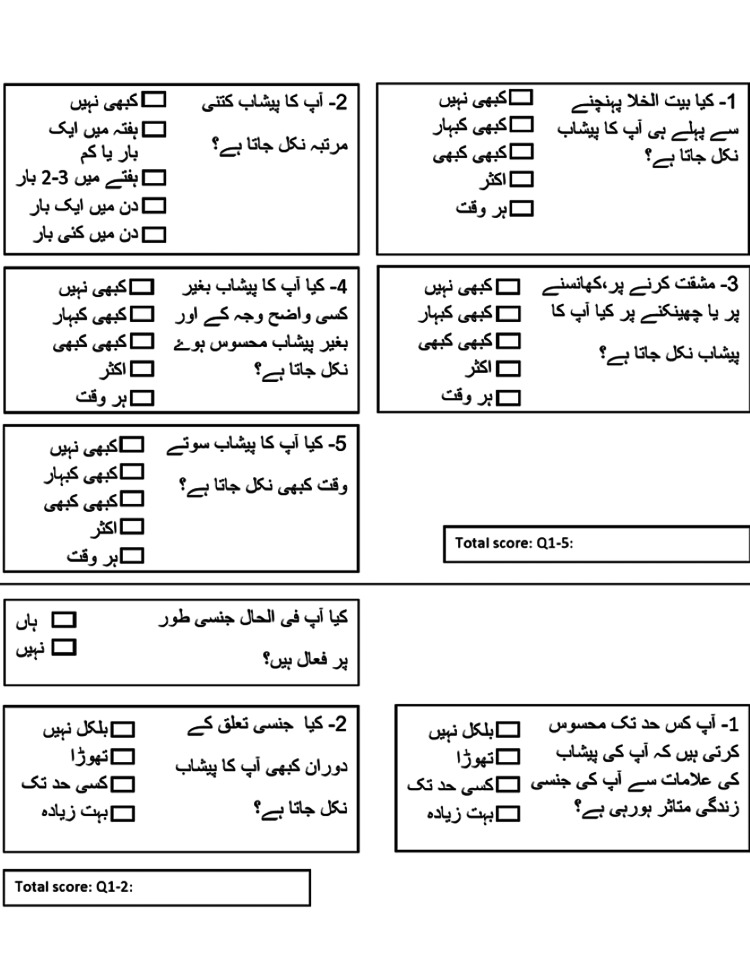
Urdu questionnaire: page 2.

**Figure 3:**
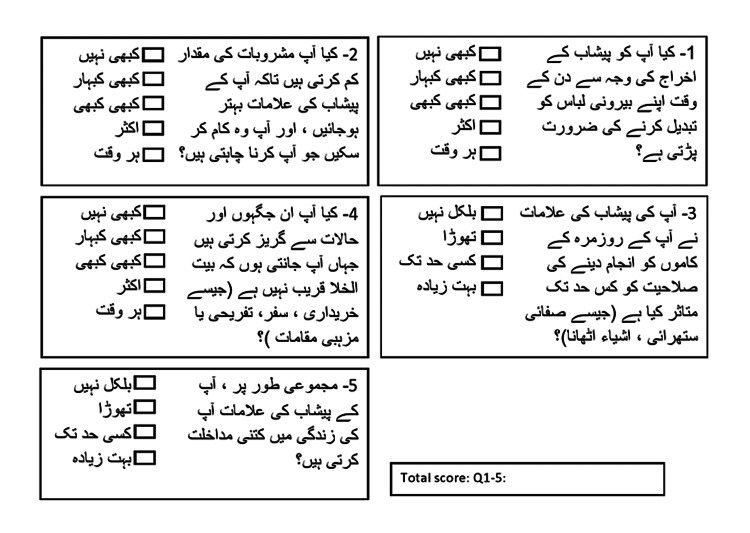
Urdu questionnaire: page 3.

## Appendices

Urdu Questionnaire
